# Optical Coherence Tomography Reveals New Insights into the Accommodation Mechanism

**DOI:** 10.1155/2015/510459

**Published:** 2015-07-06

**Authors:** Mahmoud Mohamed Farouk, Takeshi Naito, Kayo Shinomiya, Hiroshi Eguchi, Khulood Mohammed Sayed, Toshihiko Nagasawa, Takashi Katome, Yoshinori Mitamura

**Affiliations:** ^1^The Department of Ophthalmology and Visual Neuroscience, Institute of Health Biosciences, The University of Tokushima Graduate School, 3-18-15 Kuramoto-cho, Tokushima 770-8503, Japan; ^2^The Department of Ophthalmology, Sohag Faculty of Medicine, Sohag University, Sohag 82524, Egypt

## Abstract

*Purpose*. To evaluate the movement of the anterior and posterior lens poles during naturally stimulated accommodation in children using anterior segment optical coherence tomography (OCT). *Methods*. This is a prospective, observational, noncomparative case series including 18 eyes of nine children. Analysis of the anterior segment in the accommodated and unaccommodated state (with cycloplegia) was done using anterior segment OCT. The main outcome measures were the position of the anterior and posterior lens poles (in relation to the cornea) and lens thickness (LT). *Results*. A Statistically significant forward movement of the anterior lens pole and backward movement of the posterior lens pole with an increase in LT were found during accommodation (*P* < 0.001). There was no significant difference between the degree of movement of the anterior lens pole and the posterior lens pole during accommodation (*P* = 0.944). *Conclusions*. Anterior segment OCT provides a rapid noncontact method for studying accommodation in children. The backward movement of the posterior lens pole during accommodation nearly equals the forward movement of its anterior pole. These data minimize the theoretical hydraulic effect of the vitreous during accommodation, adding more support to the capsular theory of Helmholtz.

## 1. Introduction

Accommodation is a complex phenomenon by which the eye can change its refractive power to focus on near and distant objects clearly. The importance of understanding the mechanism of accommodation has made it an interesting field of study during the last two centuries. Several hypotheses regarding its mechanism have been proposed and all are in agreement that accommodation occurs by rounding of the crystalline lens to increase its refractive power [1, 2].

The anterior segment optical coherence tomography (OCT) is considered a user-friendly method to study accommodation. It is a simple, rapid, noncontact method with high image quality and is available with software capable of calculating distance and angle [3]. Other methods used previously in studying accommodation have some disadvantages which may affect the reliability of these studies [3]. One of the most widely used methods to study the crystalline lens* in vivo* is the slit lamp Scheimpflug photographic technique. This method obtains measurements along an optical slit, which may be varied by changes in the axis of the eye during stimulation of accommodation. On the other hand, accommodation is compromised by mydriasis with topical mydriatics and it does not allow the eye under study to be stimulated to accommodate naturally, with the other eye being stimulated instead [3, 4].

Ultrasonography is another method considered in the evaluation of ocular changes during accommodation by A-scan and ultrasound biomicroscopy (UBM). It can visualize the structures behind the iris with a good image quality. However, it is not suitable for studying accommodation in children as it is a contact method and needs a gel or water bath to be placed between the cornea and probe, which may also distort the anterior segment. Again, as with the slit lamp method, it does not allow stimulation of natural accommodation in the eye under examination [3].

The aim of this study was to measure the movement of the anterior and posterior lens poles in relation to the cornea during naturally stimulated accommodation in children using Casia SS-1000 anterior segment OCT (TOMEY Corporation Japan, Nagoya, Japan).

## 2. Patients and Methods

### 2.1. Study Population

Participants were recruited from the outpatient clinic of the Department of Ophthalmology at Tokushima University Hospital and also included volunteers who were relatives of hospital staff. Parents of all participants were provided with information about the study and written informed consent was obtained. The study adhered to the tenets of the Declaration of Helsinki. Ethics Committee approval was obtained.

Inclusion criteria were (1) children aged from 4 to 12 years and (2) spherical refraction from −3.00 to +1.00 diopter (D). Exclusion criteria were (1) any ocular pathology which might affect accommodation or cause deformation of the anterior segment anatomy; (2) any previous intraocular surgery; and (3) poor visual acuity or poor cooperation which may interfere with fixation on the target of the OCT equipment. Twenty-seven children were examined for participation in this study, and 9 (18 eyes) met the inclusion criteria.

### 2.2. Measurements

Manifest refraction and decimal best-corrected visual acuity (BCVA) were measured for all participants. Assessment by anterior segment OCT was done in the accommodated state first and then in the unaccommodated state after the administration of cycloplegic eye drops. All assessments and measurements were carried out by the same examiner (MF). The examiner was not masked to the patient accommodative status because the pupil was constricted in the accommodated state and dilated in the unaccommodated (cycloplegic) state.

**Figure 1:**
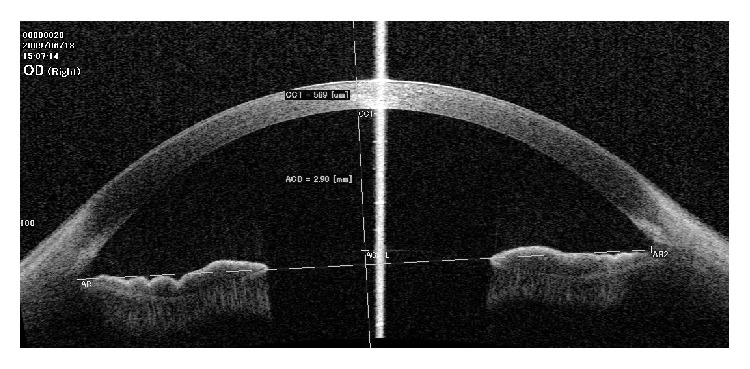
Anterior segment optical coherence tomography (OCT) image focused on the anterior chamber. Measurement of the central anterior chamber depth (ACD) is taken along the central vertical line of the anterior chamber, which is slightly temporal to the fixation line.

**Figure 2:**
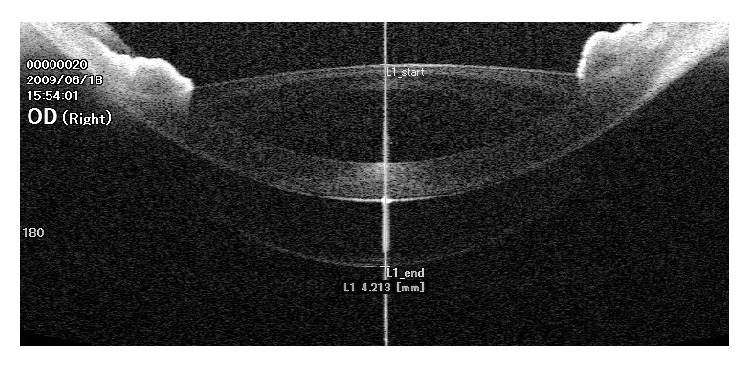
Anterior segment optical coherence tomography (OCT) image focused on the crystalline lens. Measurement of the central lens thickness is taken along the fixation line. The image of the cornea is inverted and superimposed on the image of the crystalline lens.

Measurements in the accommodated state were obtained by using the accommodation mode of Casia SS-1000 OCT which operates by defocusing the fixation target by adding a negative power lens (i.e., the fixation target will appear to be at a near distance to stimulate accommodation). We considered pupillary miosis observed during fixation on the fixation target as an indicator that the child is exerting accommodation. We adjusted the accommodation mode to −6 D taking into consideration the correction of the spherical equivalent of manifest refraction, so the accommodation power was fixed (i.e., 6 D) for all subjects. Two images were obtained: the first was focused on the anterior chamber (Figure 1) to measure the central anterior chamber depth (ACD); the second image was focused more posteriorly on the crystalline lens to measure the central lens thickness (LT); in this case the cornea appears as an inverted ghost image superimposed on the image of the crystalline lens (Figure 2). Both images were obtained along the horizontal cross section and centered on the fixation line which appears as a high reflective line nearly perpendicular to the cornea. All images were corrected by the Casia SS-1000 OCT software which can readjust the image dimensions by eliminating the distortions induced by the corneal optical transmission difference.

Measurement of the central ACD was done using the Casia SS-1000 OCT software. At first the examiner localized and marked the sinus of the angle of the anterior chamber in both sides, and then the software drew a transverse line between them, representing the horizontal plane of the anterior chamber, and a vertical line perpendicular to its center. The examiner localized and marked the intersection between this central vertical line and the anterior lens capsule, and then the software calculated the distance between this point and another point at the intersection of the central vertical line and the posterior corneal surface. This distance was considered the central ACD (Figure 1).

Measurement of the central LT was done through the pupil as the iris pigments will prevent the infrared beam from penetrating the iris and will interfere with OCT assessment of the crystalline lens peripheral to the pupil [4]. Measurement was obtained along the fixation line between 2 points at its intersection with the anterior and posterior lens capsule (Figure 2).

After taking measurements during accommodation, 1% cyclopentolate eye drops were used for mydriasis and cycloplegia. Two drops were instilled in each eye followed by another two drops after 5 minutes and measurements were taken 25 minutes later. Measurements in the unaccommodated (cycloplegic) state were obtained in the same manner used for the measurements in the accommodated state described above but with the accommodation mode turned off (i.e., the fixation target will appear to be at a far distance to relax accommodation).

Regarding the position of the lens poles in the accommodated and unaccommodated states, measurements were calculated as follows: the anterior lens pole position was considered to be equal to the central ACD. The position of the posterior lens pole was considered to be equal to the sum of the central ACD and central LT.

The main outcome measures of this study were* the position* of the lens poles (anterior and posterior) in relation to the posterior corneal surface and the central LT in accommodated and unaccommodated states as well as* the change* in position of the lens poles (anterior and posterior) and the changes in central LT during accommodation. All measurements were in millimeters (mm).

### 2.3. Statistical Analysis

All analyses were performed using SPSS for Windows version 9.0 (SPSS, Inc., Chicago, IL). The main outcome measures were expressed as mean (±standard deviation). Comparison of means was performed by paired Student's *t*-test. A *P* value <0.05 was considered significant.

## 3. Results

This study included 18 eyes of nine children, whose demographic characteristics are shown in Table 1.

**Figure 3:**
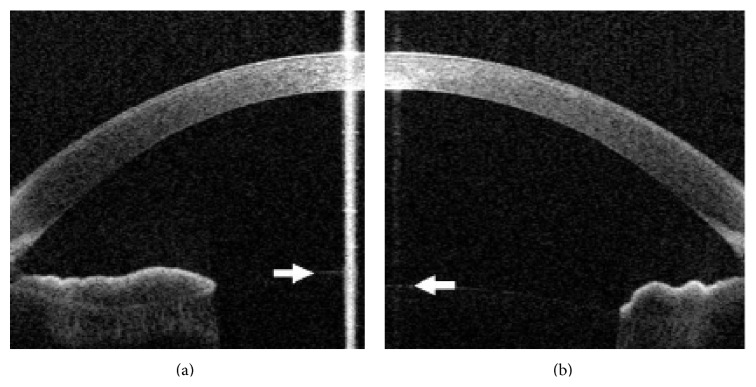
Anterior segment optical coherence tomography (OCT) image focused on the anterior chamber in the accommodated (a) and unaccommodated (b) state, showing forward movement of the anterior lens pole (arrows) with accommodation.

The anterior lens pole position, the mean central lens thickness, and the posterior lens pole position showed a statistically significant difference between the accommodated and the unaccommodated state. Forward movement of the anterior lens pole and backward movement of the posterior lens pole with accommodation were noted in all eyes (Figure 3). There was no statistically significant difference between the extent of movement of the anterior lens pole and the extent of movement of posterior lens pole (*P* = 0.944). All results are summarized in Table 2.

## 4. Discussion

The capsular theory of accommodation proposed by Helmholtz in 1855 is still the most accepted theory in spite of being questioned by Tscherning in 1900 and later by Schachar in 1992 [5–7]. The Helmholtz theory postulated that accommodation occurs by contraction of the ciliary muscles, releasing the resting tension on the zonular fibers, which in turn releases the outward directed equatorial tension on the lens capsule, allowing the elasticity of the lens to make it more round [8].

The hydraulic suspension theory proposed by Coleman in 1986 suggested that the vitreous support to the posterior lens surface has a hydraulic effect on the lens by the increased vitreous pressure during contraction of the ciliary muscles. Coleman described the movement and the change of curvature of the posterior lens surface during accommodation to be minimal, and if the capsular mechanism is true, there should actually be more bulging of the thinner posterior capsule than of the thicker anterior capsule [9–11]. This was supported by the observations of Koretz et al. on changes of lens position in aniridic rhesus monkey eyes during induced accommodation evaluated by slit lamp Scheimpflug photographs and A-scan ultrasonography, where the posterior lens surface remained fixed relative to the cornea [12]. Koretz and Handelman described the role of the lens capsule during accommodation as a force distributor, spreading the tension applied by the zonules evenly over the surface of the underlying lens material, with the accommodative process requiring a contribution from vitreous support [13]. On the other hand, if the vitreous has the main role in accommodation, vitrectomy surgery would result in marked impairment of accommodation. This was not the rule in the study of Nishide et al., which reported that the power of accommodation after lens-sparing vitrectomy is preserved [14].

With the development of new examination methodologies such as anterior segment OCT, some of these observations should be revised. Baikoff et al. used this method to study human natural accommodation in a 19-year-old albino where they found that the anterior and posterior capsule moved forward and backward, respectively [4].

The anterior lens pole was found in our study to move forward by 0.21 ± 0.15 mm during accommodation. This was proved by other investigators as Konradsen et al., who compared accommodations in patients with Marfan's syndrome and in a healthy control group by anterior segment OCT. Their control group showed changes in anterior chamber depth from 3.17 ± 0.34 mm during cycloplegia to 2.93 ± 0.32 during accommodation [15]. Baikoff et al. also performed a dynamic analysis of the anterior segment during accommodation by anterior segment OCT and estimated that the anterior lens pole moves forward by 0.3 mm with 10 D of accommodation [3].

UBM was used by Kaluzny to measure the anterior movement of the crystalline lens during accommodation in children. He found that the anterior pole moves forward but to variable degrees depending on refraction: 0.144 ± 0.14 mm in emmetropic eyes, 0.071 ± 0.13 mm in myopic eyes, and 0.242 ± 0.16 mm in hyperopic eyes [16]. It was not possible to compare these results with our results because of the mismatch in age and refraction.

Different studies estimated the increase in lens thickness per diopter of accommodation using different techniques (anterior segment OCT, slit lamp Scheimpflug photography, and ultrasonography). However, results are not usually correlated with each other because of the difference in accommodative stimuli, lack of accurate recording of the accommodative response, variation of resolution of the different techniques, and differences in assumed refractive index and speed of sound [17]. Our absolute values of lens thickness (during the accommodated and unaccommodated states) may be altered by anterior segment OCT as it assumed a fixed refractive index [18], but this would not significantly affect the change in lens thickness during accommodation which is our considered outcome.

It is universally accepted that, during accommodation, the anterior lens pole moves forward and the lens thickness increases, but whether the posterior lens pole moves forward or backward or remains stable is still unclear [4, 10, 12, 16]. Our study demonstrated that the posterior lens pole moves backward during accommodation by a mean of 0.21 ± 0.17 mm which is not significantly different from the forward movement of the anterior lens pole (*P* = 0.944). These results are contrary to the slit lamp studies on the rhesus monkey eye by Koretz et al., who found that the posterior lens surface is fixed relative to the cornea during accommodation [12]. Also, our results do not agree totally with the Coleman model of the accommodative mechanism and his hydraulic theory as he demonstrated that there is much greater forward movement of the anterior surface than the corresponding backward movement of the posterior surface. Coleman explained this by the effect of vitreous support to the back surface of the lens, which he considered to have a stronger role in accommodation than the role of the elasticity of the capsule [9, 10]. Our data gives more support to the capsular theory of Helmholtz but at the same time does not exclude the vitreous support concept of Coleman, because if it has no effect, the thinner posterior capsule would protrude more than the thicker anterior capsule. Future studies could evaluate the posterior lens pole movement during accommodation in phakic vitrectomized eyes of children or young adults (i.e., nonpresbyopic individuals) to clarify the role of vitreous in accommodation.

Data obtained by the Kaluzny UBM study of movement of the crystalline lens during accommodation revealed a significant effect of refractive status on the movement of the posterior pole. It was found to be fixed in position in emmetropia and move backward in myopia and forward in hypermetropia [16]. In our study, the effect of refractive status was not evaluated because of the small size of the sample and narrow range of variation of the refractive status of the participants (0.75 to −2.75 D), but we found that all cases showed backward movement irrespective of the refractive status. Future large sample studies could use anterior segment OCT to detect the effect of refraction on posterior pole movement during accommodation.

We recommend repeating the study using different types of imaging techniques with the same subjects (i.e., Pentacam and anterior segment OCT) and comparing the results to evaluate the repeatability of the anterior segment OCT.

One of the weak points in our study is that we are not sure that the eye is accommodating during imaging. Actually we can say that it is an assumption. The pupillary miosis is not a sure indicator of accommodation, because it might be related to the light of the internal fixation target. Recently, Neri et al. studied the accommodation process in normal eyes using Casia SS-1000 OCT by the accommodation mode with different amplitudes (0, 3.0, 6.0, and 9.0 diopters) and concluded that it is an effective method to study accommodation [18]. On the other hand, the measurements in the unaccommodated state in our study cannot be considered the true changes occurring during the physiological relaxation of accommodation because we obtained the images under the effect of cycloplegia which is not the physiological state of the eye.

In conclusion, the current study demonstrates that anterior segment OCT provides a rapid noncontact method to study accommodation in children. Results suggest that the increased thickness of the crystalline lens during accommodation in children is associated with backward movement of the posterior lens pole nearly equal to the forward movement of the anterior lens pole. These data minimize the hydraulic effect of the vitreous on the lens during accommodation, adding more support to the capsular theory of Helmholtz.

## Figures and Tables

**Table 1:** Demographic characteristics of the study sample. D: dioptre; BCVA: best corrected visual acuity.

Mean age ± SD (range), y	7.6 ± 2.8 (4–10)
Gender	
Male, number	8
Female, number	1
Spherical equivalent refraction, D	−0.72 ± 1.09 (range, −2.75 to +0.75)
Snellen BCVA	1.18 ± 0.26 (range, 0.80 to 1.50)

**Table 2:** Measurements in accommodated and unaccommodated state^a^.

	Accommodated	Unaccommodated	*P* value	Amount of change
Position of anterior lens pole^b^	2.890 ± 0.29	3.103 ± 0.26	<0.001	0.213 ± 0.15
Central lens thickness	4.609 ± 0.27	4.186 ± 0.24	<0.001	0.422 ± 0.20
Position of posterior lens pole^b^	7.498 ± 0.15	7.289 ± 0.18	<0.001	0.209 ± 0.17

^a^All values are in millimeters and are expressed as mean ± standard deviation.

^b^Position of the anterior and posterior lens pole was measured in relation to the posterior corneal surface.

## References

[1] Kaufman P. L., William M. H. (1992). Accommodation and presbyopia: neuromuscular and biophysical aspects. *Adler's Physiology of the Eye: Clinical Application*.

[2] Werner L., Trindade F., Pereira F. (2007). Physiology of accommodation and presbyopia. *Arquivos Brasileiros de Oftalmologia*.

[3] Baikoff G., Lutun E., Ferraz C., Wei J. (2004). Static and dynamic analysis of the anterior segment with optical coherence tomography. *Journal of Cataract and Refractive Surgery*.

[4] Baikoff G., Lutun E., Wei J., Ferraz C. (2004). Anterior chamber optical coherence tomography study of human natural accommodation in a 19-year-old albino. *Journal of Cataract and Refractive Surgery*.

[5] Lee D. B. (2002). Error tolerance in Helmholtzian accommodation. *Ophthalmology*.

[6] Glasser A., Kaufman P. L. (1999). The mechanism of accommodation in primates. *Ophthalmology*.

[7] Schachar R. A., Bax A. J. (2001). Mechanism of accommodation. *International Ophthalmology Clinics*.

[8] von Helmholtz H. *Helmholtz's Treatise on Physiological Optics*.

[9] Coleman D. J. (1986). On the hydraulic suspension theory of accommodation. *Transactions of the American Ophthalmological Society*.

[10] Coleman D. J. (1970). Unified model for accommodative mechanism. *American Journal of Ophthalmology*.

[11] Barraquer R. I., Michael R., Abreu R., Lamarca J., Tresserra F. (2006). Human lens capsule thickness as a function of age and location along the sagittal lens perimeter. *Investigative Ophthalmology and Visual Science*.

[12] Koretz J. F., Bertasso A. M., Neider M. W., True-Gabelt B., Kaufman P. L. (1987). Slit-lamp studies of the rhesus monkey eye: H. changes in crystalline lens shape, thickness and position during accommodation and aging. *Experimental Eye Research*.

[13] Koretz J. F., Handelman G. H. (1982). Model of the accommodative mechanism in the human eye. *Vision Research*.

[14] Nishide T., Kato Y., Mizuki N. (2006). Power of accommodation following lens-preserving vitrectomy. *Rinsho Ganka*.

[15] Konradsen T. R., Koivula A., Kugelberg M., Zetterström C. (2009). Accommodation measured with optical coherence tomography in patients with Marfan’s syndrome. *Ophthalmology*.

[16] Kaluzny B. J. (2007). Anterior movement of the crystalline lens in the process of accommodation in children. *European Journal of Ophthalmology*.

[17] Richdale K., Bullimore M. A., Zadnik K. (2008). Lens thickness with age and accommodation by optical coherence tomography. *Ophthalmic and Physiological Optics*.

[18] Neri A., Ruggeri M., Protti A., Leaci R., Gandolfi S. A., Macaluso C. (2015). Dynamic imaging of accommodation by swept-source anterior segment optical coherence tomography. *Journal of Cataract & Refractive Surgery*.

